# Therapeutic Hypothermia after Cardiac Arrest Attenuates Hindlimb Paralysis and Damage of Spinal Motor Neurons and Astrocytes through Modulating Nrf2/HO-1 Signaling Pathway in Rats

**DOI:** 10.3390/cells12030414

**Published:** 2023-01-26

**Authors:** Ji Hyeon Ahn, Tae-Kyeong Lee, Dae Won Kim, Myoung Cheol Shin, Jun Hwi Cho, Jae-Chul Lee, Hyun-Jin Tae, Joon Ha Park, Seongkweon Hong, Choong-Hyun Lee, Moo-Ho Won, Yang Hee Kim

**Affiliations:** 1 Department of Physical Therapy, College of Health Science, Youngsan University, Yangsan, Gyeongnam 50510, Republic of Korea; 2 Department of Biomedical Science and Research Institute for Bioscience and Biotechnology, Hallym University, Chuncheon, Gangwon 24252, Republic of Korea; 3 Department of Biochemistry and Molecular Biology, Research Institute of Oral Sciences, College of Dentistry, Kangnung-Wonju National University, Gangneung, Gangwon 25457, Republic of Korea; 4 Department of Emergency Medicine, School of Medicine, Kangwon National University, Chuncheon, Gangwon 24289, Republic of Korea; 5 Department of Neurobiology, School of Medicine, Kangwon National University, Chuncheon, Gangwon 24341, Republic of Korea; 6 Bio-Safety Research Institute, College of Veterinary Medicine, Chonbuk National University, Iksan, Chonbuk 54596, Republic of Korea; 7 Department of Anatomy, College of Korean Medicine, Dongguk University, Gyeongju, Gyeongbuk 38066, Republic of Korea; 8 Department of Surgery, Kangwon National University Hospital, School of Medicine, Kangwon National University, Chuncheon, Gangwon 24289, Republic of Korea; 9 Department of Pharmacy, College of Pharmacy, Dankook University, Cheonan, Chungnam 31116, Republic of Korea

**Keywords:** asphyxia, body temperature, neuroinflammation, neuroprotection, reactive gliosis, return of spontaneous circulation, spinal gray matter

## Abstract

Cardiac arrest (CA) and return of spontaneous circulation (ROSC), a global ischemia and reperfusion event, lead to neuronal damage and/or death in the spinal cord as well as the brain. Hypothermic therapy is reported to protect neurons from damage and improve hindlimb paralysis after resuscitation in a rat model of CA induced by asphyxia. In this study, we investigated roles of nuclear factor erythroid 2-related factor 2 (Nrf2) and heme oxygenase-1 (HO-1) in the lumbar spinal cord protected by therapeutic hypothermia in a rat model of asphyxial CA. Male Sprague-Dawley rats were subjected to seven minutes of asphyxial CA (induced by injection of 2 mg/kg vecuronium bromide) and hypothermia (four hours of cooling, 33 ± 0.5 °C). Survival rate, hindlimb motor function, histopathology, western blotting, and immunohistochemistry were examined at 12, 24, and 48 h after CA/ROSC. The rats of the CA/ROSC and hypothermia-treated groups had an increased survival rate and showed an attenuated hindlimb paralysis and a mild damage/death of motor neurons located in the anterior horn of the lumbar spinal cord compared with those of the CA/ROSC and normothermia-treated groups. In the CA/ROSC and hypothermia-treated groups, expressions of cytoplasmic and nuclear Nrf2 and HO-1 were significantly higher in the anterior horn compared with those of the CA/ROSC and normothermia-treated groups, showing that cytoplasmic and nuclear Nrf2 was expressed in both motor neurons and astrocytes. Moreover, in the CA/ROSC and hypothermia-treated group, interleukin-1β (IL-1β, a pro-inflammatory cytokine) expressed in the motor neurons was significantly reduced, and astrocyte damage was apparently attenuated compared with those found in the CA/ROSC and normothermia group. Taken together, our results indicate that hypothermic therapy after CA/ROSC attenuates CA-induced hindlimb paralysis by protecting motor neurons in the lumbar spinal cord via activating the Nrf2/HO-1 signaling pathway and attenuating pro-inflammation and astrocyte damage (reactive astrogliosis).

## 1. Introduction

The arterial supply of the spinal cord is supplied by the vertebral arteries at the cervical level, the dorsal intercostal arteries at the thoracic level, and the lumbar arteries at the lumbar level of the spinal cord [1]. Spinal cord ischemia, i.e., interruption of blood supply to the spinal cord, can happen in a variety of situations, including CA [2,3] and endovascular aortic surgery [4,5].

Because the gray matter of the spinal cord has a rich capillary network [6], the spinal motor neurons have been reported to be susceptible to transient spinal cord ischemia. Delayed neuronal death of motor neurons located in the anterior gray matter is observed after spinal cord ischemia in rabbits [7] and mice [8], as well as in the anterior gray matter after CA in rats [9]. As a result of damage to or death of the spinal motor neurons, hindlimb motor dysfunction occurs [10].

It has been reported that spinal cord injury leading to cellular damage is closely associated with free radical production, glutamate excitotoxicity, lipid peroxidation, inflammation, and glial cell proliferation in the spinal gray matter [11]. In animal models of spinal cord injuries induced by CA [12] and spinal cord impactor [13], treatments of hypothermia [12] and medication (imatinib, a tyrosine kinase inhibitor) [13] can improve hindlimb motor outcome and save spinal motor neurons after injuries. These treatments are linked to significant reductions in oxidative stress and inflammatory cytokines, and marked increases in antioxidant production [12,13,14,15].

Nrf2 is a ubiquitous master transcription factor that upregulates cytoprotective proteins and antioxidant defense gene expression [16]. HO-1, an isoform of inducible heme oxygenase (HO), shows an important role in regulating barrier function and inflammation in the acute phase of an injured spinal cord [10]. The upregulation of the Nrf2/HO-1 signaling pathway after hypothermic therapy in rat models of CA-induced brain ischemia and reperfusion (IR) damage [17] and CA-induced renal IR injury [18] is involved in the protection of hippocampal pyramidal cells and renal cortical tissues, respectively. However, studies on the roles of Nrf2 and HO-1 in therapeutic hypothermia after CA-induced spinal cord IR injury are lacking.

Therefore, using a rat model of spinal cord IR injury following CA induced by asphyxia, we investigated the effects of therapeutic hypothermia after CA on paraplegia (loss of movement in both hindlimbs), motor neuron damage, and Nrf2/HO-1 expression over time in the lumbar level of the ischemic spinal cord. In addition, changes in IL-1β (a pro-inflammatory cytokine) and astrocytes (glial cells responsible for trophic and metabolic support of neurons) were examined.

## 2. Materials and Methods

### 2.1. Experimental Protocol and Animals

The protocol of all animal experiments was approved (approval no. KW-200113-1) from by the Institutional Animal Care and Use Committee, an Ethics Committee of Kangwon National University. Animal care and use described in the protocol was done according to “The Guide for the Care and Use of Laboratory Animals” (The National Academies Press, 8th Ed., 2011). Efforts were also made to minimize animal suffering during the whole experiment.

Male Sprague-Dawley rats, weighing 320–350 g, were purchased from Central Lab Animal Inc. (Seoul, South Korea). The rats were housed in pathogen-free environment under standard conditions: controlled at about 24 °C of room temperature and 55% of relative humidity, with a steady dark and light cycle every 12 h and freely accessible food and water.

For this experiment, four groups were divided as follows: (1) The sham-normothermia (NT) group (*n* = 44) received a sham CA/ROSC operation and was controlled at normothermia (37 ± 0.5 °C) for four hours after the sham operation; (2) the CA-NT group (*n* = 28) received a CA/ROSC operation and was controlled at NT for four hours after CA/ROSC; (3) the sham-hypothermia (HT) group (*n* = 44) received a sham CA/ROSC operation and was controlled at HT (33 ± 0.5 °C) for four hours after CA/ROSC; and (4) the CA-HT group (*n* = 28) received CA/ROSC operation and was controlled at HT for four hours after CA/ROSC. The rats (*n* = 6 for histology and 5 for western blotting, respectively) in the two CA/ROSC groups were sacrificed at 12, 24, and 48 h after CA/ROSC. The rats (*n* = 6 for histology and 5 for western blotting, respectively) of the two sham groups were sacrificed at 0 and 48 h after CA/ROSC to reduce the numbers of the rats, and the results of the two sham groups were not significantly different. Therefore, the results of the sham group were presented at 48 h after CA/ROSC.

### 2.2. Induction of Asphyxial CA/ROSC and Therapeutic Hypothermia

**Figure 1:**
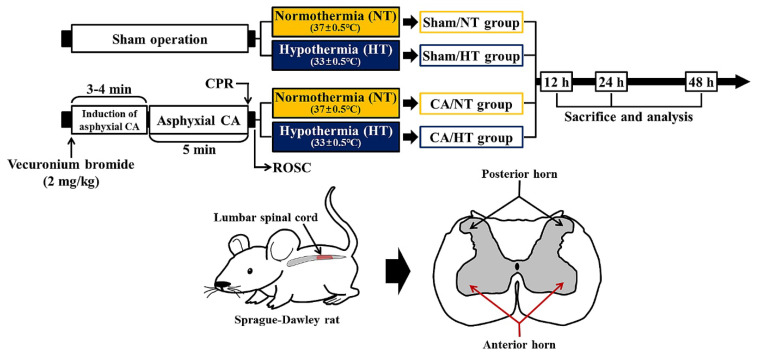
Experimental timeline with design. CA, CPR, and ROSC were performed at designated times. Therapeutic HT was done after ROSC for four hours. Sacrifice was done for analyses in the anterior horn of the lumbar spinal cord at 12, 24, and 48 h after CA/ROSC.

As shown in Figure 1, the operation of CA/ROSC was performed, as described in previous studies [19,20,21], with some modifications. Briefly, as shown in Figure 1, all rats were fasted overnight, anesthetized with 2.5% isoflurane (4 L/min) and endotracheally intubated with a 14-gauge cannula through a tracheotomy under mechanical ventilation using a ventilator (Harvard Apparatus, Holliston, MA, USA).

For mean arterial pressure (MAP), a PE-50 catheter (ADInstruments Ltd., Sydney, Australia) was canulated into the left femoral artery and monitored using MLT 1050/D (ADInstruments Ltd., Sydney, Australia). Blood sampling was also done at the femoral artery. Another catheter was canulated in the right femoral vein for the delivery of drugs and fluids. Peripheral oxygen saturation (SpO_2_) was monitored using a pulse oximetry (Nonin Medical Inc., Plymouth, MN, USA) on the left foot. Electrocardiogram (ECG) was monitored using electrocardiographic probes (GE healthcare, Milwaukee, WI, USA) connected on all four limbs with three electrode leads (GE healthcare, Chicago, IL, USA), respectively.

At five minutes after stabilization and collection of baseline data, to induce asphyxial CA, 2 mg/kg of vecuronium bromide (Reyon Pharmaceutical, Seoul, South Korea) was injected through the right femoral vein. Three to four minutes later, complete CA was defined by confirming less than 25 mmHg of MAP and pulseless electrical activity on the ECG. To control the body temperature during the surgery, temperature was controlled at normothermia (37 ± 0.5 °C) using a thermometric blanket (Harvard Apparatus, Holliston, MA, USA) and TR-100 rectal temperature probe (Fine Science Tools, Foster City, CA, USA).

Immediately, ROSC was carried out as follows: Cardiopulmonary resuscitation (CPR) was initiated after five minutes of CA. Briefly, under chest compression and ventilation oxygen was supplied using the ventilator, and 0.005 mg/kg of epinephrine (Dai Han Pharm, Seoul, South Korea) and 1 meq/kg of sodium bicarbonate (Daewon Pham) was intravenously injected. In this experiment, the manual chest compression (300/min) for ROSC was done until ECG activity was shown and MAP reached 60 mmHg [22,23].

Hypothermic therapy after ROSC was provided by surface cooling with a cooling blanket, ice packs, isopropyl alcohol wipes, and an electrical fan according to published protocols [9,24]. Hypothermia in the sham-HT and CA-HT groups was maintained at 33 ± 0.5 °C for four hours, and normothermia in the sham/NT and CA/NT groups was maintained at 37 ± 0.5 °C using a heating pad and lamps.

### 2.3. Motor Function Evaluation

A neurological test for motor function of the hindlimbs was performed based on the Tarlov Scale [25] on day 1 after CA/ROSC. Motor function of the hindlimbs was graded as 0 = complete paraplegia (no voluntary movement); 1 = noticeable movement at joints (slight movement); 2 = good movement at joints (but no ability to stand); 3 = ability to stand and walk (but inability to walk normally); 4 = complete recovery (normal movement).

### 2.4. Fluoro Jade B (FJB) Histofluorescence

In this experiment, we used histofluorescence with FJB (a marker for neuronal degeneration) to examine neuronal death (loss) in the lumbar level of the spinal cord following CA/ROSC. For the preparation of spinal cord tissues, as previously described [9], all rats were profoundly anesthetized by intraperitoneal injection of 200 mg/kg of sodium pentobarbital (JW Pharmaceutical, Seoul, South Korea). Under the anesthesia, the rats were rinsed transcardially with 100 mM phosphate-buffered saline (pH 7.4) and fixed with 4% paraformaldehyde (in 100 mM phosphate buffer, pH 7.4). The lumbar spinal cords were obtained from the spinal canal, fixed for six hours, and infiltrated with 25% sucrose for cryoprotection for eight hours. Thereafter, the lumbar spinal cord tissues were serially sectioned into 25-µm-thick sections in a Leica cryostat (Wetzlar, Germany).

To carry out FJB histofluorescence, as previously described [26,27], the sections were immersed in 0.06% potassium permanganate for eight minutes and then incubated in 0.0004% FJB (Histochem, Jefferson, AR, USA) for 20 min. Immediately, the sections were washed out, placed on slide warmer (45 ± 5 °C) to be reacted. Finally, the sections were covered with DPX (Sigma Aldrich, St. Louis, MO, USA).

### 2.5. Immunohistochemistry

Following antibodies were used in this study: choline acetyltransferase (ChAT) to detect cholinergic motor neurons, Nrf2 and HO-1 to investigate Nrf2/HO-1 signaling pathway, IL-1β to examine proinflammation, and glial fibrillary acidic protein (GFAP) to detect astrocytes in the lumbar spinal cord.

Immunohistochemical staining was conducted according to a published method [28] with modifications. Briefly, the spinal cord sections, which are described above, were immersed in 0.3% hydrogen peroxide (H_2_O_2_) for 20 min and then in 5% normal donkey serum for 30 min. After washing, the sections were incubated in each diluted primary antibody overnight at 4 °C as follows: rabbit anti-ChAT (1:200; Chemicon, Temecula, CA, USA), rabbit anti-Nrf2 (1:500; Proteintech, Rosemont, IL, USA), rabbit anti-HO-1 (1:500; Abcam, Cambridge, UK), rabbit anti-IL-1β (1:200; Abcam), and mouse anti-GFAP (1:800; Abcam). After washing, the sections were transferred to biotinylated horse anti-mouse or goat anti-rabbit IgG (diluted 1:200; Vector, Burlingame, CA, USA) for two hours, and followed by a streptavidin peroxidase complex (diluted 1:200; Vector) for one hour. Thereafter, the sections were washed and visualized with 0.2% 3, 3′-diaminobenzidine tetrahydrochloride. Finally, the sections were covered with DPX (Sigma Aldrich, St. Louis, MO, USA).

### 2.6. Double Immunofluorescence

To identify the cell types of cytoplasmic and/or nuclear expression of Nrf2, double immunofluorescence staining was conducted using rabbit anti-Nrf2 (1:500; Proteintech)/mouse anti-GFAP (1:800; Abcam), by referring to a published method [29]. The spinal cord sections obtained at 24 h after CA/ROSC were used as follows. The sections were incubated in the mixture of antisera for seven hours at 4 °C. Next, the sections were washed and incubated in the mixture of FITC- or Cy3-conjugated donkey anti-rabbit or anti-mouse IgG (1:200; Jackson ImmunoResearch Inc., West grove, PA, USA) for two hours at room temperature. The double immunoreaction was observed with confocal MS from the LSM510 META NLO (Carl Zeiss, Göttingen, Germany). Afterwards, the sections were covered with DPX (Sigma Aldrich).

### 2.7. Western Blotting

To investigate the Nrf2/HO-1 signaling pathway in the lumbar spinal cord, western blotting was conducted according to published methods [13,30] with modifications. Briefly, the rats were profoundly anesthetized by intraperitoneal injection of 200 mg/kg of sodium pentobarbital, and their lumbar spinal cords were removed to homogenize and ultracentrifuge for collecting supernatants. Isolation of nuclear fractions of the lumbar spinal cords was conducted using an extraction kit (Thermos Scientific, Rockford, IL, USA) by referring to the manufacturer’s instructions and a published paper [30]. A Bicinchoninic Acid (BCA) Protein Assay Kit obtained from Pierce Chemical (Thermos Scientific) was used to quantify the final protein concentration. Protein samples (40 μg) were loaded, separated by SDS-PAGE, and transported to nitrocellulose membranes obtained from Pall Corporation (Pittsburgh, PA, USA). Thereafter, the membranes were blocked with 5% bovine serum albumin for two hours at room temperature, followed by incubating with primary antibodies: rabbit anti-Nrf2 (110 kDa, 1:1000; Abcam), rabbit anti-HO-1 (33 kDa, 1:1000; Abcam), rabbit anti-β-actin (42 kDa, 1:5,000; Abcam), and specific nuclear rabbit anti-lamin B (67 kDa, 1:1,500; Santa Cruz Biotechnology, CA, USA) overnight at 4 °C. Finally, the membranes were incubated with peroxidase-conjugated goat anti-rabbit IgG (1:4,500; Santa Cruz Biotechnology) and visualized with an enhanced chemiluminescence (ECL) kit obtained from GE Healthcare Life Sciences (Chalfont, UK).

### 2.8. Analyses of Data

For quantitative analyses of neuronal loss (death) or protection, six sections per rat were selected with 140 μm intervals. ChAT- and FJB-positive (^+^) cells or neurons were counted as previously described [31]. Briefly, the digital images of ChAT^+^- and FJB^+^ cells were captured and obtained at a magnification of 10× using the BX53 upright microscope (Olympus, Tokyo, Japan), equipped with an Olympus DP72 digital camera and Olympus cellSens Standard image capture software. The mean number of ChAT^+^ and FJB^+^ cells were assessed by averaging the total number of cells counted in a 500 μm^2^ area in the center of the anterior horn using Image J 1.59 software (United States National Institute of Health, Bethesda, MD, USA).

For the quantification of Nrf2, HO-1, IL-1β, and GFAP immunoreactive structures, the digital image of each structure was taken using the above-mentioned method and analyzed by referring to a published paper [32]. Briefly, each immunostained structure was captured in a 500 μm^2^ in the center of the anterior horn and evaluated as relative optical density (ROD) using Adobe Photoshop 8.0 (San Jose, CA, USA) and NIH Image software 1.59 (NIH, Bethesda, MD, USA). ROD was calibrated as % compared with the sham-NT group (100%).

For quantitative analyses of protein levels obtained from the western blotting, the scanning of Nrf2, HO-1, and nuclear Nrf2 bands was performed by the ChemiDoc Imaging System (Bio-Rad Laboratories Inc., Hercules, CA, USA), and densitometry analysis was conducted using Scion Image software (Ver 4.0; Scion Corp., Frederic, MD, USA). The ROD of Nrf2, HO-1, and nuclear Nrf2 proteins were calibrated with the corresponding expression rate of β-actin or lamin B1, and normalized to that in the sham-NT group.

### 2.9. Statistical Analyses

All the experiments were performed in a completely randomized manner. Sample size was calculated using G*Power 3 (Erdfelder, Faul, & Buchner, 2007) [33], with an alpha error of 0.05 and a power of >80%, resulting in a minimum of two rats per group. In the present study, data are expressed as mean ± SD. All data obtained here were analyzed through SPSS software version 18.0 (SPSS Inc., Chicago, IL, USA). The assumptions of normality were assessed using Shapiro-Wilk’s test. The homogeneity of variances was verified using Lavene’s test. The significance of differences between the groups was assessed through an independent t-test, a one-way analysis of variance (ANOVA), and post-hoc Tukey’s test. A *p*-value less than 0.05 was typically considered to be statistically significant.

## 3. Results

### 3.1. Survival Rate and Physiological Characteristics

In this study, the cumulative survival rate was evaluated using Kaplan-Meier analysis. All rats in the sham-NT and sham-HT groups survived until 2 days after the sham CA operation. In the CA-NT group, the cumulative survival rates were 72.7%, 46.3%, and 7.7% at 12, 24, and 48 h, respectively, after CA/ROSC. However, in the CA-HT group, the cumulative survival rate has increased: 90.9%, 74.4%, and 37.2% at 12, 24, and 48 h, respectively, after CA/ROSC.

There were no statistically significant differences between the groups regarding baseline characteristics, including body weight, SpO_2_ and MAP (no data shown): this finding was similar to that obtained in a previous study [34].

### 3.2. Hindlimb Motor Function

**Figure 2:**
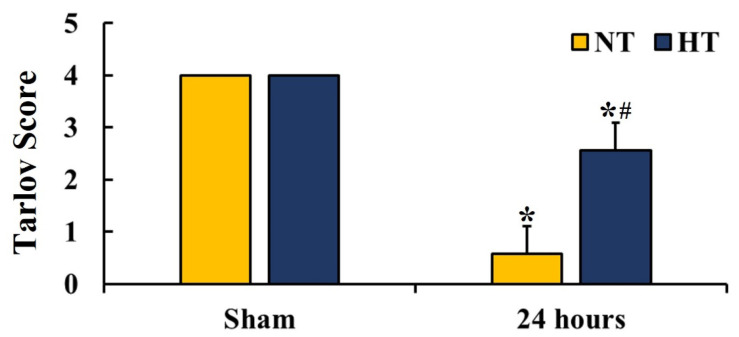
Hindlimb motor function evaluation in the sham-NT, sham-HT, CA-NT, and CA-HT groups at 24 h after CA/ROSC. The rats of the CA-NT and CA-HT groups show hindlimb motor dysfunction, but the CA-HT group shows significantly milder motor dysfunction when compared with the CA-NT group. A high score indicates good motor function. The bars indicate the means ± SD (*n* = 6, respectively; * *p* < 0.05 vs. each sham group, # *p* < 0.05 vs. corresponding CA-NT group).

Hindlimb motor function was examined with a modified Tarlov score at 24 h after CA/ROSC (Figure 2). The rats of the sham-NT and sham-HT groups showed normal hindlimb motor function 24 h after CA/ROSC. The rats of the CA-NT group revealed severe hindlimb motor dysfunction (14.3% of the sham), whereas the rats of the CA-HT group showed mild motor dysfunction in the hindlimb (64.3% of the sham; *t*(10) = 6.474, *p* < 0.0001).

### 3.3. Protection of Motor Neurons

#### 3.3.1. ChAT-Immunoreactive (ChAT^+^) Neurons

**Figure 3:**
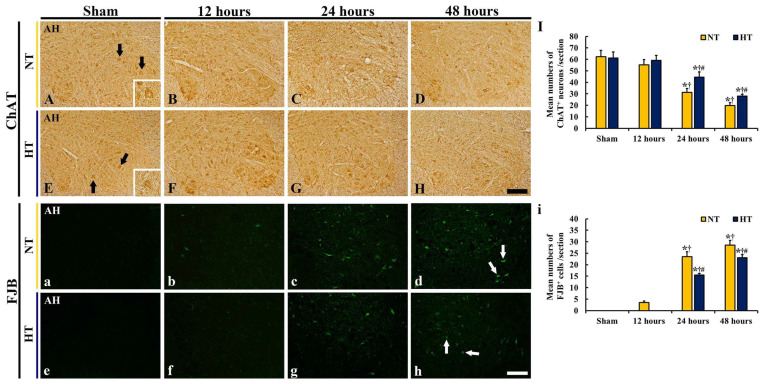
(**A**–**H** and **a**–**h**) ChAT immunohistochemistry (A-H) and FJB fluorescence (**a**–**h**) in the anterior horn of the sham-NT (**A**, **a**), sham-HT (**E**, **e**), CA-NT (**B**–**D**, **b**–**d**), and CA-HT (**F**–**H**, **f**–**h**) groups at 12 (**B**,**F**,**b**,**f**), 24 (**C**,**G**,**c**,**g**), and 48 (**D**,**H**,**d**,**h**) hours after CA/ROSC. In both CA-NT and CA-HT groups, ChAT^+^ motor neurons (black arrows) are significantly reduced at 24 and 48 h after CA/ROSC; however, the numbers found in the CA-HT group are significantly higher than those in the CA-NT group. The numbers of FJB^+^ motor neurons (white arrows) are significantly increased in the CA-NT group at 24 and 48 h after CA/ROSC, but, in the CA-HT group, FJB^+^ motor neurons are significantly low in number compared with the CA-NT group. AH, anterior horn. Scale bar = 200 μm. (**I** and **i**) Mean numbers of ChAT^+^ (**I**) and FJB^+^ (**i**) motor neurons in the anterior horn. The bars indicate the means ± SD (*n* = 6, respectively; * *p* < 0.05 vs. each sham group; † *p* < 0.05 vs. previous time-point group; # *p* < 0.05 vs. corresponding CA-NT group).

ChAT immunohistochemistry was performed in the lumbar spinal cord to detect motor neurons, which are located in the anterior horn, the general somatic efferent for the hindlimbs, and contain ChAT (Figure 3A–H). There was a significant difference in number of ChAT^+^ neurons between the groups (*F*(7,40) = 53.684, *p* < 0.0001).

In the sham-NT group, many ChAT^+^ motor neurons were clearly shown throughout the anterior horn (Figure 3A). In the CA-NT group, the numbers of ChAT^+^ motor neurons were slightly reduced (88.7% of the sham-NT group) at 12 h after CA/ROSC (Figure 3B,I), and at 24 and 48 h after CA/ROSC, the numbers of ChAT^+^ motor neurons were significantly decreased (50.2% and 31.8% of the sham-NT group, respectively) (Figure 3C,D,I).

In the sham-HT group, the distribution of ChAT^+^ motor neurons was similar to that found in the sham-NT group (Figure 3E,I). In the CA-HT group, the numbers of ChAT^+^ motor neurons at 12 h after CA/ROSC were not significantly different from those of the sham-HT group (Figure 3F,I), and at 24 and 48 h after CA/ROSC, the decrease in the numbers of ChAT^+^ motor neurons was attenuated (71.4% and 45.0% of the sham-NT group, respectively) compared with those of the CA-NT group (Figure 3G–I).

#### 3.3.2. FJB-Positive (FJB^+^) Cells

To examine the degeneration or death of motor neurons, FJB (a fluorescent marker of neuronal degeneration or death) fluorescence staining was conducted in all groups (Figure 3a–h). There was a significant difference in the number of FJB^+^ cells between the groups (*F*(7,40) = 1150.6, *p* < 0.0001).

In the sham-NT group, no FJB^+^ cells were shown in the anterior horn (Figure 3a). In the CA-NT group, a few FJB^+^ motor neurons appeared (3.5/section) in the anterior horn 12 h after CA/ROSC (Figure 3b,i). At 24 and 48 h after CA/ROSC, the numbers of FJB^+^ motor neurons were significantly increased (23.4/section and 28.3/section, respectively) after CA/ROSC (Figure 3c,d,i).

In the sham-HT group, no FJB^+^ motor neurons were also detected in the anterior horn (Figure 3e). In the CA-HT group, FJB^+^ motor neuron cells were also not found at 12 h after CA/ROSC (Figure 3f,i). At 24 and 48 h after CA/ROSC, the numbers of FJB^+^ motor neurons were significantly reduced (15.6/section and 23.1/section, 65.9% and 80.7% of CA-NT group, respectively) (Figure 3g–i).

### 3.4. Changes in Nrf2 and HO-1 Expressions

#### 3.4.1. Nrf2 and HO-1 Protein Levels

**Figure 4:**
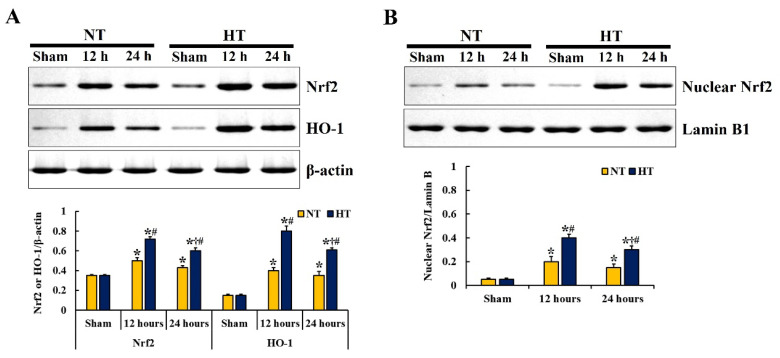
Representative western blot images and relative protein expression levels of Nrf2 and HO-1 (**A**), and nuclear Nrf2 (**B**) in the lumbar spinal cord of the sham-NT, sham-HT, CA-NT, and CA-HT groups at 12 and 24 h after CA/ROSC. In the CA-HT group, the levels of Nrf2, HO-1, and nuclear Nrf2 proteins are significantly increased at 12 and 24 h after CA/ROSC compared with the CA-NT group. The bars indicate the means ± SD (*n* = 5, respectively; * *p* <0.05 vs. each sham group; † *p* < 0.05 vs. previous time-point group; # *p* < 0.05 vs. corresponding CA-NT group).

To investigate the molecular mechanisms of the protective effects of hypothermia against CA-induced spinal cord injury, changes in the levels of Nrf2 and HO-1 proteins in the lumbar spinal cord were examined at 12 and 24 h after CA/ROSC (Figure 4). There was a significant difference in Nrf2 (*F*(5,12) = 138.71, *p* < 0.0001) and HO-1 (*F*(5,12) = 212.79, *p* < 0.0001) proteins between the groups.

In the sham-NT and sham-HT groups, no significant differences in Nrf2 and HO-1 protein levels were found (Figure 4A). In the CA-NT group, the levels of Nrf2 and HO-1 proteins were significantly increased at 12 h (1.4-fold and 2.6-fold of the sham-NT group, respectively) and stayed high at 24 h (1.2-fold and 2.3-fold of the sham-NT group, respectively) after CA/ROSC (Figure 4A). In the CA-HT group, the levels of Nrf2 and HO-1 proteins were further significantly increased after CA/ROSC (2.1-fold and 5.3-fold of the sham-NT group at 12 h, and 1.7-fold and 4.0-fold of the sham-NT group at 24 h after CA/ROSC) compared with the CA-NT group (Figure 4A).

In addition, the nuclear translocation of Nrf2 was examined after CA/ROSC (Figure 4B). There was a significant difference in nuclear Nrf2 protein levels between the groups (*F*(5,12) = 77.667, *p* < 0.0001). No significant difference in nuclear Nrf2 protein levels between the sham-NT and sham-HT groups was found (Figure 4B). In the CA-NT group, a significant accumulation of nuclear Nrf2 protein was shown at 12 and 24 h after CA/ROSC (4.2-fold and 2.8-fold of the sham-NT group, respectively) (Figure 4B). In the CA-HT group, nuclear Nrf2 protein levels were further significantly increased at 12 and 24 h after CA/ROSC (8.4-fold and 6.2-fold of the sham-NT group, respectively) compared with the CA-NT group (Figure 4B).

#### 3.4.2. Nrf2 Immunoreactivity

**Figure 5:**
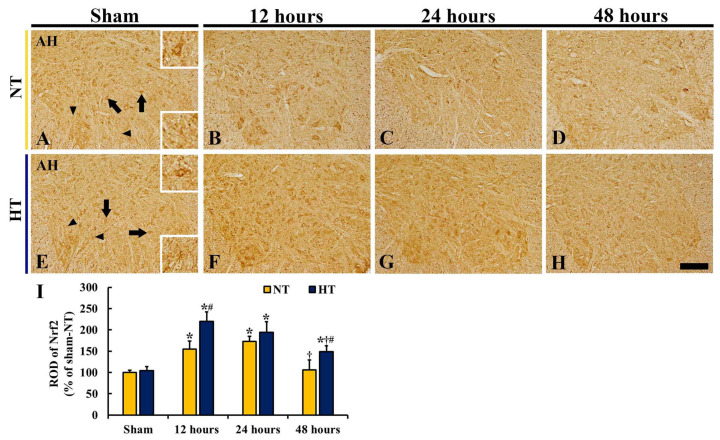
(**A**–**H**) Nrf2 immunohistochemistry in the anterior horn of the sham-NT (**A**), CA-NT (**B**,**C**), sham-HT (**E**), and CA-HT (**F**–**H**) groups at 12 (**B**,**F**), 24 (**C**,**G**), and 48 (**D**,**H**) hours after CA/ROSC. Nrf2 immunoreactivity is shown in the cytoplasm of motor neurons (arrows and upper right box) and glia-like cells (arrowheads and lower right box) in the anterior horn of the sham-NT group. In the CA-NT group, Nrf2 immunoreactivity is increased at 12 h and highest at 24 h. In the CA-HT group, Nrf2 immunoreactivity is higher than in the CA-NT group, showing that the immunoreactivity is highest at 12 h after CA/ROSC. AH, anterior horn. Scale bar = 200 μm. (I) ROD of Nrf2 immunoreactive structures in the anterior horn. The bars indicate the means ± SD (*n* = 6, respectively; * *p* < 0.05 vs. each sham group; ^†^*P* < 0.05 vs. previous time-point group; # *p* < 0.05 vs. corresponding CA-NT group).

To investigate the expression pattern and subcellular location of Nrf2 in the anterior horn of the lumbar spinal cord, Nrf2 immunohistochemistry was conducted (Figure 5). There was a significant difference in Nrf2 immunoreactivity between the groups (*F*(7,40) = 53.684, *p* < 0.0001).

In the sham-NT group, Nrf2 immunoreactivity was observed in the cytoplasm of motor neurons and glia-like cells located in the anterior horn (Figure 5A): Nrf2 subcellular location in the motor neurons was confirmed by a morphological feature according to our previous studies using neuronal nuclei (NeuN) immunohistochemistry and Cresyl Violet staining [9,12]. In the CA-NT group, the ROD of Nrf2^+^ structures was significantly increased at 12 and 24 h after CA/ROSC (155.2% and 172.4% of the sham-NT group, respectively) compared with the sham-NT group (Figure 5B,C,I), and at 48 h after CA/ROSC, the ROD of Nrf2^+^ structures was decreased (105.8% of the sham-NT group) (Figure 5D,I).

In the sham-HT group, Nrf2 immunoreactivity shown in the anterior horn was similar to that in the sham-NT group (Figure 5E,I). In the CA-HT group, Nrf2 immunoreactivity was significantly high at all points in time after CA/ROSC compared with the CA-NT group, showing that the ROD peaked at 12 h after CA/ROSC (219.6% of the sham-NT group) and gradually decreased, but it was higher than the CA-NT group (194.1% at 24 h and 148.8% at 48 h compared with the sham-NT group) (Figure 5G–I).

#### 3.4.3. Nrf2 Immunofluorescence

**Figure 6:**
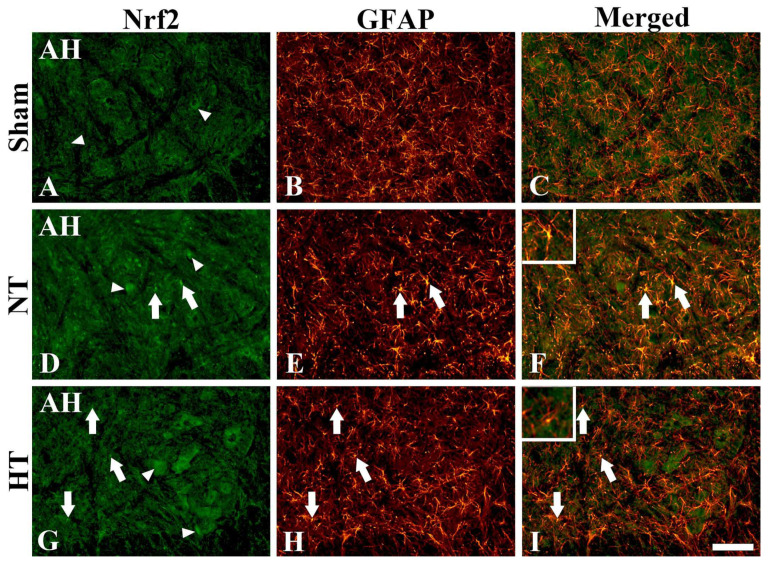
Double immunofluorescence for Nrf2 (green; **A**,**D**,**G**), GFAP (red; **B**,**E**,**H**), and merged (**G**,**F**,**I**) images in the anterior horn of the sham-NT or HT (**A**–**C**), CA-NT (**D**–**F**), and CA-HT (**G**–**I**) groups at 24 h after CA/ROSC. Nrf2 immunoreactivity is mainly found in the cytoplasm of motor neurons (arrowheads) and astrocytes in all groups, showing that, in the CA-NT and CA-HT groups, Nrf2 immunoreactivity is found in both the cytoplasm and nucleus of some motor neurons. Note that arrows indicate Nrf2^+^ astrocytes, which show the nuclear pattern of Nrf2 immunoreactivity in the CA-NT and CA-HT groups. Upper left boxes show the enlargement of an astrocyte with nuclear Nrf2 immunoreactivity. AH, anterior horn. Scale bar = 50 μm.

As described above, Nrf2 immunoreactivity was shown in the motor neurons and glia-like cells of all sham and CA groups at 24 h after CA/ROSC (Figure 5). For motor neurons, as shown in Figure 6A, Nrf2 immunoreactivity in the sham group was clearly found in the cytoplasm of the motor neurons; however, as shown in Figure 6D,G. Nrf2 immunoreactivity in the CA-NT and CA-HT groups was found in the cytoplasm and nucleus of the motor neurons. For glial cells, to identify the cell type of the Nrf2^+^ glial cells, double immunofluorescence staining was conducted with Nrf2 and GFAP (a marker for astrocytes) (Figure 6). Nrf2 immunoreactivity was found in small cells (glia-like cells) in all groups (Figure 6A,D,G). Double immunofluorescence revealed that the Nrf2^+^ glial cells turned out to be GFAP^+^ astrocytes, some of which showed the nuclear pattern of Nrf2 immunoreactivity only in the CA-NT and CA-HT groups (Figure 6D–I).

#### 3.4.4. HO-1 Immunoreactivity

**Figure 7:**
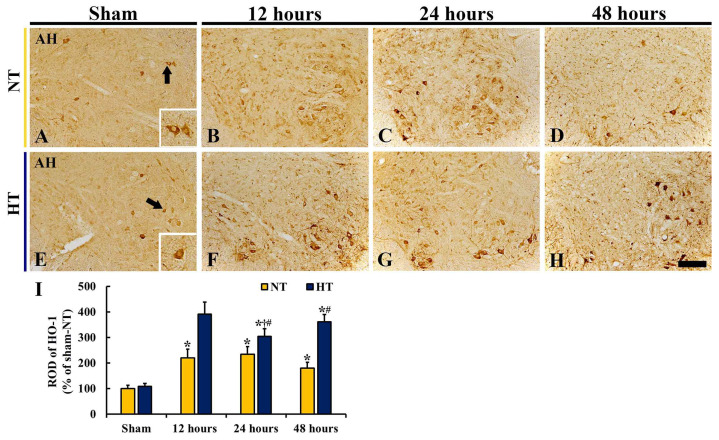
(**A**–**H**) HO-1 immunohistochemistry in the anterior horn of the sham-NT (**A**), CA-NT (**B**–**D**), sham-HT (**E**), and CA-HT (**F**–**H**) groups at 12 (**B**,**F**), 24 (**C**,**G**), and 48 (**D**,**H**) hours after CA/ROSC. HO-1 immunoreactivity is shown in motor neurons (arrows and lower right box) in both sham groups. In the CA-NT group, HO-1 immunoreactivity peaks at 24 h and decreases at 48 h due to death of motor neurons after CA/ROSC. The CA-HT group, HO-1 immunoreactivity peaks at 12 h after CA/ROSC and is significantly higher at all points in time after CA/ROSC than the CA-NT group. AH, anterior horn. Scale bar = 200 μm. (I) ROD of HO-1^+^ structures. The bars indicate the means ± SD (*n* = 6, respectively; * *p* < 0.05 vs. each sham group; † *p* < 0.05 vs. previous time-point group; # *p* < 0.05 vs. corresponding CA-NT group).

There was a significant difference in HO-1 immunoreactivity between the groups (*F*(7,40) = 100.75, *p* < 0.0001). HO-1 immunoreactivity in the sham-NT group was strongly found in the cytoplasm of many motor neurons in the anterior horn (Figure 7A): HO-1 subcellular location in the motor neurons was confirmed by a morphological feature according to our previous studies using NeuN immunohistochemistry and Cresyl Violet staining [9,12]. In the CA-NT group, HO-1 immunoreactivity of motor neurons was significantly increased after CA/ROSC (ROD: 219.1% at 12 h, 234.3% at 24 h, and 179.4% at 48 h after CA/ROSC compared with the sham-NT group) (Figure 7B,C,I), showing that, at 48 h after CA/ROSC, the numbers of HO-1^+^ motor neurons were apparently decreased due to their death.

In the sham-HT group, HO-1 immunoreactivity shown in the anterior horn was similar to that in the sham-NT group (Figure 7E). In the CA-HT group, intense HO-1 immunoreactivity was shown after CA/ROSC, showing that the ROD of HO-1^+^ structures was significantly higher (391.5% at 12 h, 304.3% at 24 h, and 360.4% at 48 h after CA/ROSC) than the CA-NT (Figure 7F–I).

### 3.5. Changes in IL-1β

**Figure 8:**
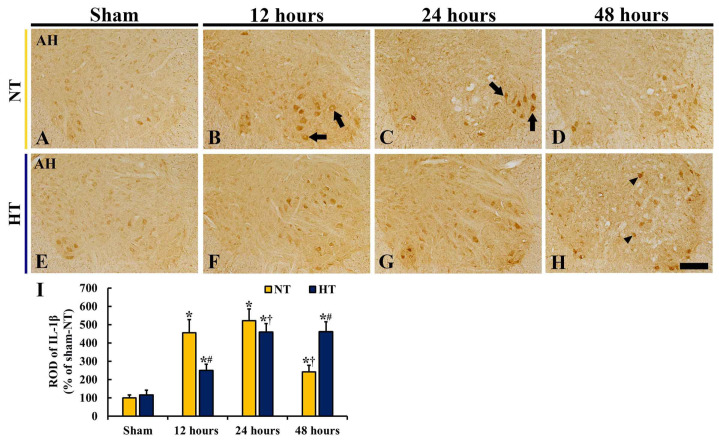
(**A**–**H**) IL-1β immunohistochemistry in the anterior horn of the sham-NT (**A**), CA-NT (**B**–**D**), sham-HT (**E**), and CA-HT (**F**–**H**) groups at 12 (**B**,**F**), 24 (**C**,**G**), and 48 (**D**,**H**) hours after CA/ROSC. IL-1β immunoreactivity is found in motor neurons. In the CA-NT group, IL-1β immunoreactivity is significantly increased (arrows) at 12 and 24 h after CA/ROSC and then reduced at 48 h after CA/ROSC. In the CA-HT group, increased IL-1β immunoreactivity at 12 and 24 h after CA/ROSC is lower than in the CA-NT group, but increased IL-1β immunoreactivity at 48 h (arrowheads) is apparently higher than in the CA-NT group. AH, anterior horn. Scale bar = 200 μm. (I) ROD of IL-1β^+^ structures. The bars indicate the means ± SD (*n* = 6, respectively, * *p* < 0.05 vs. each sham group; † *p* < 0.05 vs. previous time-point group; # *p* < 0.05 vs. corresponding CA-NT group).

Changes in proinflammation in the anterior horn after CA/ROSC were examined using immunohistochemistry for IL-1β (a pro-inflammatory cytokine) (Figure 8). There was a significant difference in IL-1β immunoreactivity between the groups (*F*(7,40) = 91.333, *p* < 0.0001).

In the sham-NT group, IL-1β immunoreactivity was found in the cytoplasm of motor neurons (Figure 8A). In the CA-NT group, IL-1β immunoreactivity in the motor neurons was strongly increased at 12 and 24 h after CA/ROSC (ROD: 456.1% and 521.1% of the sham-NT group, respectively) compared with the sham-NT group (Figure 8B,C,I); however, at 48 h after CA/ROSC, the IL-1β immunoreactivity was reduced (241.5% of the sham-NT group) due to motor neuron damage (Figure 8D,I).

In the sham-HT group, IL-1β immunoreactivity in the motor neurons was similar to that in the sham-NT group (Figure 8E,I). In the CA-HT group, IL-1β immunoreactivity was significantly increased at 12 and 24 h after CA/ROSC, but the ROD was low (54.8% and 87.9%, respectively, of corresponding CA-NT group) compared with the CA-NT group (Figure 8F–I). At 48 h after CA/ROSC, IL-1β immunoreactivity was maintained (ROD: 461.7% of the sham-NT group) (Figure 8H,I): at this point in time, the motor neurons were saved from ischemic injury.

### 3.6. Changes in Reactive Astrocytes

**Figure 9:**
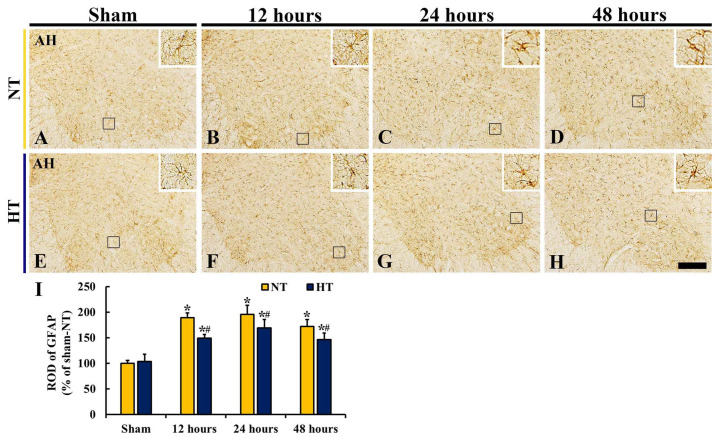
(**A**–**H**) GFAP immunohistochemistry in the anterior horn of the sham-NT (**A**), CA-NT (**B**–**D**), sham-HT (**E**), and CA-HT (**F**–**H**) groups at 12 (**B**,**F**), 24 (**C**,**G**), and 48 (**D**,**H**) hours after CA/ROSC. In both CA-NT and CA-HT groups, GFAP^+^ astrocytes react to ischemic insult (enlarged cell bodies and thickened processes); GFAP immunoreactivity of the CA-HT group is lower than that of the CA-NT group. Note that damages of GFAP^+^ processes (boxes of **C**,**D**,**H**) is apparent at 24 and/or 48 h after CA/ROSC. AH, anterior horn. Scale bar = 200 μm. (I) ROD of GFAP^+^ structures. The bars indicate the means ± SD (*n* = 6, respectively; * *p* < 0.05 vs. each sham group; # *p* < 0.05 vs. corresponding CA-NT group).

GFAP immunohistochemistry was conducted to detect astrocytes and examine changes in astrocyte response to CA/ROSC-induced ischemic insult in the anterior horn (Figure 9). There was a significant difference in GFAP immunoreactivity between the groups (*F*(7,40) = 55.545, *p* < 0.0001).

In the sham-NT group, GFAP^+^ astrocytes were evenly distributed in the anterior horn, and as a resting form, they had long and thin processes with small-sized cytoplasm (Figure 9A). In the CA-NT group, GFAP^+^ astrocytes were reactive to ischemic insult induced by CA/ROSC: they had hypertrophied cytoplasm with thickened processes after CA/ROSC (Figure 9B–D), showing that the ROD of GFAP^+^ structures was significantly increased at 12, 24, and 48 h after CA/ROSC (188.7%, 195.5%, and 172.1% of the sham-NT group, respectively) compared with the sham-NT group (Figure 9B–D,I). In particular, GFAP^+^ astrocytes at 24 and 48 h after CA/ROSC were apparently damaged: the processes were destroyed and lost (Figure 9C,D).

In the sham-HT group, the distribution and morphology of GFAP^+^ astrocytes was not different form the sham-NT (Figure 9E). In the CA-HT group, GFAP^+^ astrocytes also reacted to ischemic insult, showing that the ROD of GFAP^+^ structures was significantly increased at 12, 24, and 48 h after CA/ROSC (148.8%, 169.1%, and 145.9% of the sham-NT group, respectively); however, GFAP immunoreactivity was lower than that shown in the CA-NT group at each point in time after CA/ROSC (Figure 9F–I). In addition, at 24 and 48 h after CA/ROSC, damage of the processes of GFAP^+^ astrocytes was attenuated compared with those of the CA-NT group (Figure 9G,H).

## 4. Discussion

In this experiment, we examined the effects of therapeutic hypothermia on survival rate, hindlimb paraplegia, and damage/death of motor neurons located in the lumbar spinal cord in a rat model of asphyxial CA/ROSC. In addition, we investigated changes in the Nrf2/HO-1 signaling pathway, proinflammation, and reactive astrogliosis in the anterior horn.

For spinal cord ischemia in mice, it has been reported that spinal cord ischemia is developed by clamping the aorta and causes paraplegia with increased mortality depending on the degree of ischemia [8,35]. Namely, mice with spinal cord ischemia for 11 min, but not for nine minutes develop acute and complete paraplegia and show a mortality rate of 21% within 24 h [35]. In addition, mice with mild ischemia duration (less than five minutes) all survive and show a delayed neurological deficit of the hindlimbs, whereas mice with severe ischemia (eight to 12 min) result in immediate and progressive paraplegia, with a survival rate of 0 % within 36 h after the ischemia [8]. For spinal cord ischemia induced by CA/ROSC in rats that received asphyxial CA for five minutes showed a gradual reduction in survival rate (65.3% at 24 h and 4.3% at 48 h after AC/ROSC) [36]. In our current study, CA/ROSC under NT resulted in severe paraplegia (hindlimb motor dysfunction) at 24 h after CA/ROSC and caused a low survival rate of 7.7% at 48 h after CA/ROSC. However, paralysis in the ischemic rats treated with hypothermia was significantly attenuated, and the survival rate increased to 37.2% at 48 h after CA/ROSC. The results of previous and present studies indicate that prolonged spinal cord ischemia induced by systemic circulation interruption such as CA or aortic occlusion leads to severe neurological deficits in the hindlimbs and increased mortality, but therapeutic hypothermia increases the survival rate and attenuates paraplegia.

Studies have reported that neurological motor deficits in the hindlimbs after spinal cord ischemia are due to the death/damage of motor neurons located in the lumbar levels of the spinal cord. Apoptosis of neurons in the gray matter of the lumbar levels is shown one day after 14 min of spinal cord ischemia in rats [37] and two days following 15 min of spinal cord ischemia in rabbits [7]. In addition, an extensive damage of motor neurons in the lumbar levels was shown two days after 11 min of spinal cord ischemia in mice [35]. In our current study, motor neurons located in the lumbar anterior horn of the CA-NT group contained ChAT, and their numbers were significantly decreased from 12 h after CA/ROSC; this was confirmed by the finding that FJB^+^ neurons were found 12 h after CA/ROSC. However, in the CA-HT group, the loss or damage of the lumbar motor neurons was significantly reduced from 24 h after CA/ROSC. This finding is similar to that reported in a another study, showing that one and two days after asphyxial CA, rats received an immediate hypothermia therapy, and the neurons located in the lumbar anterior horn were significantly more abundant than those of rats with CA and normothermia [12]. Taken together, CA-induced neuronal loss or damage in the lumbar anterior horn, which contains motor neurons, must be attenuated by hypothermia therapy.

It has been demonstrated that hypothermia attenuates CA-induced hippocampal morphology changes in rats and shown that the protective effect of hypothermia following CA might be related to inhibition of apoptosis and oxidative stress through, at least in part, activation of the GSK-3β/Nrf2/HO-1 pathway [17]. Based on the paper, we investigated whether the effect of neuroprotection by hypothermia after asphyxial CA/ROSC was related to the Nrf2/HO-1 signaling pathway in the spinal cord. Nrf2 is known to be a master transcription factor that upregulates antioxidant response element-mediated expressions of antioxidant enzymes and cellular protective genes: Nrf2 translocates to the nucleus under oxidative stress and binds to the antioxidant response element to upregulate antioxidant enzyme genes [38,39]. In our present analyses using western blotting and immunohistochemistry, the expressions of Nrf2 and HO-1 in both CA-NT and CA-HT groups were dramatically enhanced after CA/ROSC, showing that the levels in the CA-HT group were significantly higher than those in the CA-NT group. In addition, Nrf2 immunohistochemistry revealed that, in the sham group, Nrf2 immunoreactivity in the motor neurons and astrocytes was generally shown in their cytoplasm, but, in the CA-NT and CA-HT groups, Nrf2 immunoreactivity was found in both cytoplasm and nucleus. It has been reported that hypothermia significantly increases the immunoreactivities of superoxide dismutase, catalase, and glutathione peroxidase in the anterior horn of the lumbar spinal cord following cardiac arrest [12]. Similar to our results, it has been reported that the expression levels of Nrf2 and or HO-1 are markedly increased in the spinal cord damaged by a weight-drop method [13,40,41], showing that the levels are significantly raised after treatment of zinc ions [40], imatinib [13], and aspirin [41], resulting in increases of antioxidant enzymes (superoxide dismutase and glutathione peroxidase) and decreases in oxidative stress (malondialdehyde and reactive oxygen species), inflammatory cytokines (tumor necrosis factor-α, IL-6, IL-1β) and astrocyte activation [13,40,41]. Oxidative stress and inflammatory cytokines are critical causes of primary and secondary injuries after spinal cord injury [38,42], so alleviating oxidative stress and inflammation is considered an effective intervention in spinal cord injury. Taken together, our current findings indicate that hypothermia treatment is significantly effective in protecting spinal motor neurons from whole-body ischemia and reperfusion injury induced by asphyxial CA/ROSC via activating the Nrf2/HO-1 signaling pathway.

Most inflammatory reactions following acute brain ischemia are mediated by cytokines, which are small glycoproteins expressed by many types of cells in response to the ischemia [43]. Increased levels of proinflammatory cytokines have been detected in ischemic cortex one hour after focal brain ischemia induced by middle cerebral artery occlusion in rats [44]. IL-1β is a key immunoregulatory and proinflammatory cytokine that affects almost all cell types [45]. IL-1 has been highly implicated in the pathogenesis of ischemic cerebral damage, suggesting that IL-1β, as a major form of IL-1, contributes to ischemic injury rather than IL-1α [46]. In our present experiment, the expression of IL-1β was significantly increased in motor neurons at 12 and 24 h after CA/ROSC, but hypothermia therapy after CA/ROSC notably reduced the elevated IL-1β expression. It has been reported that the injection of proinflammatory cytokines extends infarct volume and enhances edema after focal brain ischemia in rodents, but the injection of hypertonic saline or antibodies against proinflammatory cytokines decreases brain injury [43,47]. Altogether, a neuroprotective therapy targeted to modulate cytokine-induced inflammation can be a way to prevent early deterioration in acute ischemic insults.

We, in this study, investigated changes of astrocytes (astrogliosis) in all groups on the basis of a paper reporting that, in a pathological condition, IL-1β connects with the activation and proliferation of astrocytes [48]. Many researchers have looked upon astrogliosis (astrocyte responses to damage or diseases in the CNS) as “activation of astrocytes” or “activated astrocytes”. In this paper, we used “reactive astrocytes” instead of the term “activated astrocytes” in ischemia and reperfusion injury in the CNS, because the astrocyte responses have been a controversial subject. According to previous studies, astrocyte responses reveal double-sidedness (beneficial and/or harmful effects) [49,50,51]. In ischemic stroke, reactive astrocytes exert beneficial effects, such as neuroprotective effects and restoration, and detrimental effects, such as secretion of inflammatory modulators that lead to exacerbation of an ischemic lesion [51,52]. In our current experiment, the numbers of GFAP^+^ astrocytes and GFAP immunoreactivity were markedly increased in the anterior horn after CA/ROSC, showing that, at 48 h after CA/ROSC, certain GFAP^+^ astrocytes were damaged as their processes were lost and thickened with the ends being blunt. Some papers have shown that the degeneration of astrocytes occurs in the cerebral cortex after ischemic and reperfusion, as shown in gerbils [51,53]. Altogether, reactive astrocytes (astrogliosis) in the lumbar anterior horn with ischemic injury following CA/ROSC are implicated in CA/ROSC-mediated neuronal damage or death.

Finally, we found that, in the CA-HT group, astrogliosis (GFAP^+^ astrocyte response) was apparently ameliorated in the lumbar anterior horn, and in particular, the damage of astrocyte processes was apparently attenuated by hypothermia treatment. The Nrf2 immunoreactivity was not shown in all GFAP^+^ astrocytes located in the anterior horn of both sham groups, but was clearly found in the cytoplasm and nucleus of certain GFAP^+^ astrocytes of the CA-NT and CA-HT groups by double immunofluorescence for Nrf2 and GFAP. This finding indicates that CA/ROSC induces Nrf2 activation in certain CA/ROSC-mediated astrocytes. Astrocytes closely interact with neurons for synaptic transmission and energy metabolism and support neuronal resistance to oxidative stress through Nrf2-driven genes, such as HO-1 which is activated in neurons [54,55]. It has been reported that Nrf2 levels are increased and colocalized with astrocytes in the degenerated lumbar spinal cord, and overexpression of Nrf2 in reactive astrocytes prevents apoptosis of motor neurons, resulting in increased survival of motor neurons in a rat model of amyotrophic lateral sclerosis [56]. Another study has reported that hyperactivation of Nrf2 in astrocytes protects the spinal cord from oxidative stress through suppressing the expression of 4-Hydroxynonenal (an oxidative stress marker) and pro-inflammatory cytokines, including IL-1β in mice with a GFAP-specific Kelch-like ECH-associated protein 1 (Keap1)-deletion [30]. Taken together, our findings suggest that the upregulation of Nrf2 in spinal astrocytes after CA/ROSC contributes to neuroprotection against ischemic spinal cord injury induced by CA/ROSC.

## 5. Conclusions

This study showed that CA/ROSC in rats caused very low survival rates and complete paraplegia (hindlimb motor dysfunction) at 48 h after CA/ROSC; however, therapeutic hypothermia after CA/ROSC significantly improved the CA/ROSC-induced survival rate and paraplegia. In the anterior horn of the lumbar spinal cord, motor neurons and astrocytes were damaged or lost after CA/ROSC, but therapeutic hypothermia saved or significantly attenuated lumbar motor neurons from CA/ROSC insult, showing that the expressions of Nrf2 and HO-1 were more increased in motor neurons of the ischemic rats with treated hypothermia, than the non-treated ischemic rats. In addition, proinflammation and reactive gliosis were apparently attenuated by hypothermia in the ischemic rats treated with hypothermia. Taken together, the neuroprotective effects of hypothermia after CA/ROSC were closely related to the activation of Nrf2/HO-1 signaling in spinal motor neurons, or astrocytes with attenuation of IL-1β expression and astrocyte damage. Pharmacological modulation of Nrf2/HO-1 signaling, which plays a major role in hypothermia-induced neuroprotection, could be a potential future therapeutic strategy to protect spinal neurons from ischemic injury induced by cardiac arrest, warranting further investigation. The protection of spinal neurons is associated with improved motor function of the lower extremities; in humans, this protection can improve the patient’s quality of life.

## Data Availability

The data presented in this study are available on request from the corresponding author.
